# Amperometric Immunosensor for Carbofuran Detection Based on MWCNTs/GS-PEI-Au and AuNPs-Antibody Conjugate

**DOI:** 10.3390/s130405286

**Published:** 2013-04-19

**Authors:** Ying Zhu, Yaoyao Cao, Xia Sun, Xiangyou Wang

**Affiliations:** School of Agriculture and Food Engineering, Shandong University of Technology, NO.12, Zhangzhou Road, Zibo 255049, China; E-Mails: zy_881120@126.com (Y.Z.); wenlansishu@163.com (Y.C.)

**Keywords:** amperometric immunosensor, carbofuran, gold nanoparticles-antibody conjugation, multiwall carbon nanotubes, graphene sheets-PEI-Au nanocomposites

## Abstract

In this paper, an amperometric immunosensor for the detection of carbofuran was developed. Firstly, multiwall carbon nanotubes (MWCNTs) and graphene sheets-ethyleneimine polymer-Au (GS-PEI-Au) nanocomposites were modified onto the surface of a glass carbon electrode (GCE) via self-assembly. The nanocomposites can increase the surface area of the GCE to capture a large amount of antibody, as well as produce a synergistic effect in the electrochemical performance. Then the modified electrode was coated with gold nanoparticles-antibody conjugate (AuNPs-Ab) and blocked with BSA. The monoclonal antibody against carbofuran was covalently immobilized on the AuNPs with glutathione as a spacer arm. The morphologies of the GS-PEI-Au nanocomposites and the fabrication process of the immunosensor were characterized by X-ray diffraction (XRD), ultraviolet and visible absorption spectroscopy (UV-vis) and scanning electron microscopy (SEM), respectively. Under optimal conditions, the immunosensor showed a wide linear range, from 0.5 to 500 ng/mL, with a detection limit of 0.03 ng/mL (S/N = 3). The as-constructed immunosensor exhibited notable performance features such as high specificity, good reproducibility, acceptable stability and regeneration performance. The results are mainly due to the excellent properties of MWCNTs, GS-PEI-Au nanocomposites and the covalent immobilization of Ab with free hapten binding sites for further immunoreaction. It provides a new avenue for amperometric immunosensor fabrication.

## 1. Introduction

Carbofuran (2,3-dihydro-2,2-dimethylbenzofuran-7-yl methylcarbamate) is a broad-spectrum insecticide widely used in agriculture. Electrochemical immunosensors based on the high specificity of hapten (pesticides such as carbofuran) and antibody (Ab) interactions have been used to detect or quantify a specific pesticide. Compared with conventional methods for the determination of carbofuran, electrochemical immunosensors have many advantages, including simple instrumentation, easy operation, rapid response, high sensitivity, selectivity and high compatibility with advanced nanotechnology and micromachining technologies [1–4]. In order to develop a high-performance amperometric immunosensor, signal amplification and immobilization of Ab or hapten are vital in optimizing the analytical performance characteristics, such as response, reproducibility, stability, selectivity and regeneration [5]. In recent years, with the development of nanotechnology, a variety of nanoparticles, such as multiwall carbon nanotubes (MWCNTs), graphene sheets (GS) and gold nanoparticles (AuNPs), have been widely used in the fabrication of immunosensors [6,7].

Chitosan, containing large numbers of -NH_2_ and -OH groups, has been widely used as an immobilization matrix for biosensors due to its excellent biocompatibility, nontoxicity and cheapness [8,9]. It is preferable to maintain the high biological activity of the immobilized biomolecules and then enhance the sensitivity of the immunosensor, but the chitosan film is not electrically conductive. MWCNTs have attracted a great deal of interest due to their electrical properties, large specific surface areas, high stabilities and strong adsorption properties [10,11]. Thus, in recent years MWCNTs were introduced in chitosan film to improve its electric conductivity [12].

GS, a two-dimensional carbon atom monolayer, has attracted great interest for the fabrication of electrochemical immunosensors due to its high conductivity, high surface-to-volume ratio, high elasticity and good biocompatibility [13,14]. However, the water solubility of GS limits their further application in designing biosensors because GS is hydrophobic and tends to form agglomerates in water [15]. As a result, many researchers have made efforts to increase the solubility of GS. Thus, the water-soluble polymers, such as polyvinylpyrrolidone [16], polypyrrole (PPy) [17], chitosan [18,19] and Nafion [20,21] were used as dispersants to prepare homogeneous GS solutions, while the introduction of these polymers could promote electron transfer well. Significantly, some scientists have found that graphene-based composite materials, such as, gold nanoparticles and 1-pyrenebutyric acid-functionalized grapheme [22], graphene/polyaniline nanocomposite [23], AuNPs/PDDA-G [24], AuNPs decorated graphene (AuNPs-GS) [25] and MWCNTs-GS composites [26] are a useful approach. These graphene-based composite materials have good solubility and biocompatibility, and high electrochemical stability and conductivity, due to the synergistic contribution of two or more functional components. Herein, an effective reduction approach for the fabrication of GS-PEI-Au nanocomposites is demonstrated. PEI, an amino-rich cationic polyelectrolyte, is ingeniously used as both a functional agent for GS and a reducing agent and protecting agent for the formation of Au nanoparticles [27]. Combining the two functions of PEI, we can prepare GS-PEI-Au nanocomposites through *in-situ* reduction of HAuCl_4_ by PEI adsorbed on the surface of GS. In addition, in this way we improved the solubility and conductivity of GS efficiently.

For the immobilization of Ab or hapten on the modified electrode, many reviews have reported different protocols, such as physical adsorption [28], covalent coupling [29], avidin-biotin affinity reaction [30], self-assembled monolayers, *etc.* [31]. Taking an account the advantages and methods used for nanoparticles, many researchers have prepared modified AuNPs surfaces by the direct covalent linking of the Ab to the nanoparticles and assembling them onto the electrode surface [32]. Xu *et al.* developed a gold nanorods (GNRs)-Ab conjugate in which the antibody was covalently attached to GNRs with a special spatial conformation through amide (CO-NH) bonds to produce specific sensing probes for the sensitive detection of α-fetoprotein (AFP) [33].

Therefore, here we coupled anti-carbofuran Ab covalently to AuNPs with glutathione as a spacer arm. The presence of carboxyl group at the terminal end of glutathione on the AuNPs surface allowed further modification of the surface using covalent coupling reactions. The immobilization of Ab on AuNPs was carried out through a stable covalent link between the carboxyl group on the carbon-terminal of the Ab and glutathione capped AuNPs and this process was effected by 1-ethyl-3-(3-dimethylaminopropyl) carbodiimide (EDC) and 1,6-diaminohexane (DAH). This kind of approach provided a stable Ab immobilization with free hapten binding sites for further immunoreaction without affecting the structure and function of the Ab. The AuNPs also are good for the immobilization of the Ab onto the electrode and preventing them from dissolving back into the bulk solution.

As mentioned above, we introduce a MWCNTs, GS-PEI-Au nanocomposites and AuNPs-antibody conjugate-modified amperometric immunosensor for the detection of carbofuran. The aim of this work was to develop a fast, simple, inexpensive, stable and highly sensitive immunosensor for carbofuran detection. The experimental conditions related to the performance of the fabricated immunosensor (the thickness of the GS-PEI-Au layer, the pH of the supporting electrolyte, immunoassay temperature and incubation time) were investigated in detail.

## 2. Experimental

### 2.1. Materials

Anti-carbofuran monoclonal antibody, carbofuran, bovine serum albumin (BSA, 96–99%), and EDC were all purchased from Sigma (Beijing, China). HAuCl_4_ was from Shanghai Sinopharm Chemical Reagent Co. Ltd. (Shanghai, China). GS were obtained from Nanoon Co., Ltd. (Beijing, China). MWCNTs were purchased from Xfnano Co. Ltd. (Nanjing, China). PEI (Mn = 600) were purchased from Shanghai Crystal Pure Reagent Co. Ltd. (Shanghai, China).

Carbofuran was a standard grade product and other reagents were of analytical grade and distilled water was used throughout the experiments. Anti-carbofuran monoclonal antibody was dissolved with 0.01 M phosphate buffer solution (PBS, pH 7.4) processed by high-pressure sterilization and stored at 4 °C. 0.1 M 2-(*N*-Morpholino)ethanesulfonic acid buffer (MES, pH 5.0) was filtered to remove impurities and bacteria before use. A PBS (0.1 M, pH 7.0) containing 5 mM [Fe(CN)_6_]^3−/4−^ (1:1) and 0.1 M KCl was used as the detection solution.

### 2.2. Apparatus

Cyclic voltammetry (CV) and electrochemical impedance spectroscopy (EIS) measurements were performed with a CHI 650D electrochemical workstation (Shanghai Chenhua Co., Shanghai, China). All experiments were performed with a conventional three-electrode system. A modified GCE (d = 3 mm) as the working electrode, a saturated calomel electrode (SCE) and a platinum electrode were used as reference and auxiliary electrodes, respectively. X-ray diffraction (XRD) pattern was performed on a D8 ADVANCE X-ray diffractometer (Brucker AXS, Karlsruhe, Germany). Ultraviolet and visible absorption (UV-vis) spectra were obtained on a 2550 UV-spectrophotometer (Shimadzu, Kyoto, Japan). The morphologies of the GS-PEI-Au nanocomposites and the fabrication process of the immunosensor were observed using a scanning electron microscope (SEM, S-4800, Hitachi, Tokyo, Japan).

### 2.3. Preparation of MWCNTs Nanocomposites

A 0.25 wt.% chitosan solution was prepared by dissolving chitosan in 1 wt.% acetic acid solution with magnetic stirring for about 1 h, then the pH of the solution was adjusted to pH 5.0 with a concentrated NaOH solution. MWCNTs (1 mg) was added into 0.25 wt.% chitosan solution (1 mL) and then sonicated for 2 h to afford a homogeneous solution.

### 2.4. Pretreatment of GS-PEI-Au Nanocomposites

**Figure 1.**
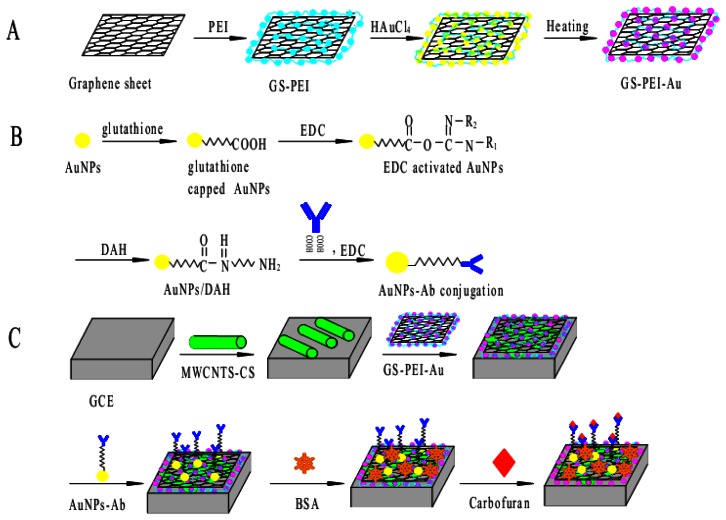
(**A**) The experimental procedure in the preparation of GS-PEI-Au nanocomposites; (**B**) Steps involved in the preparation of AuNPs-Ab conjugate; (**C**) Construction of the immunosensor.

A GS suspension was obtained by adding GS (3.0 mg) to distilled water (10 mL). The solution was sonicated for 10 min to obtain a stable, dispersed GS suspension. Then, a 1.0 M PEI aqueous solution (0.2 mL) was added into this suspension and sonicated for 10 min, followed by the addition of a 21 mM HAuCl_4_ aqueous solution (1.6 mL). This mixture was incubated in a water bath at 70 °C for 2 h. The final reaction mixture was taken out and cooled to room temperature. Then, the mixture was centrifuged at 15,000 rpm for 10 min, and the supernatant solution was discarded. This process was repeated once more, and the final sediment was dispersed in 1.5 mL of distilled water with the aid of sonication for 5 min to obtain a stable, dispersed GS-PEI-Au nanocomposite suspension. This suspension is stable for one month at room temperature. The experimental procedure for the preparation of GS-PEI-Au nanocomposites is shown schematically in Figure 1(A).

### 2.5. Preparation of AuNPs-Ab Conjugation

AuNPs-Ab conjugates were synthesised essentially according to the literature procedure [34] with some modifications. Colloidal AuNPs were synthesized by the citrate reduction method [35]. To the gold colloid solution (5 mL), glutathione (65 μM, 4 mL) was added and the mixture was stirred for 10–15 min. After the stirring was complete the mixture was centrifuged at 4,500 rpm to separate the capped AuNPs. The pellet obtained was resuspended in 1 mL of PBS.

Glutathione capped AuNPs were first activated by adding 300 μL of EDC (58 mM) in 3 mL of MES buffer (pH 5.0) and the solution was kept at 4 °C for 1.5 h on a rocker. The solution was next centrifuged at 15,000 rpm for 1 h to separate activated AuNPs and the pellet was resuspended in 0.1 M PBS. 1,6-Diaminohexane (DAH, 500 μM, 50 μL) was added to the activated AuNPs and mixed for 30 min at room temperature and the reaction mixture was again centrifuged and the pellet obtained was redissolved in PBS.

In the first step, Ab (10 μg/mL, 500 μL) was added to MES buffer (pH 5.0, 3 mL), then EDC (58 mM, 300 μL) was added and the reaction mixture was rocked on a rocker at 4 °C for 1.5 h. The activated Ab was added to AuNPs/DAH and the reaction mixture was incubated at 4 °C for 6 h. The coupled AuNPs-Ab conjugate was centrifuged at 15,000 rpm for 30 min. The AuNPs-Ab conjugation process is shown in Figure 1(B).

### 2.6. Measurement Procedure

For the measurement of carbofuran, the prepared immunosensor was first incubated in the growth solution containing different concentrations of carbofuran for 30 min and then transferred to the electrochemical cell of 5 mL 0.1 M PBS (pH 7.0) containing 5.0 mM [Fe(CN)_6_]^3−/4−^ and 0.1 M KCl as a redox probe to study the electrochemical response by CV. The carbofuran detection was based on the variation of current response (ΔI = I*p,BSA*−I*p,carbofuran*) before and after interaction between AuNPs-Ab conjugate and carbofuran, where I*p,carbofuran* is the peak current after carbofuran coupling to the immobilized AuNPs-Ab conjugate on the prepared immunosensor and I*p,BSA* is the peak current after blocking the remaining adsorption-reactive sites by BSA.

### 2.7. Preparation of Amperometric Immunosensor

The electrochemical immunosensor was prepared by the following steps: (i) before the surface modification, GCE was first polished with 0.5 and 0.03 μm alumina slurry, respectively, rinsed thoroughly with absolute alcohol and distilled water in an ultrasonic bath, and dried with N_2_ at room temperature. (ii) 10 μL of MWCNTs were initially dropped on the electrode surface, and then dried under an infrared lamp. Then 10 μL of GS-PEI-Au nanocomposites was dropped on the electrode and dried at room temperature. (iii) AuNPs-Ab conjugate was immobilized (by adsorption) onto the GS-PEI-Au/MWCNTs modified GCE. (iv) 10 μL of AuNPs-Ab conjugate solution was immobilized (by adsorption) onto the GS-PEI-Au/MWCNTs modified GCE and then the electrode was incubated at 4 °C for 12 h. (v) The possible remaining active sites on the electrode surface were blocked with 1% BSA for 30 min at room temperature. The electrode was again rinsed with distilled water to remove any residues. The resulting immunosensor was stored above 0.1 M PBS at 4 °C when not in use. The schematic illustration of the fabrication process is shown in Figure 1(C).

## 3. Results and Discussion

### 3.1. Characterization of GS-PEI-Au Nanocomposites and the Fabrication Process of the Immunosensor

**Figure 2.**
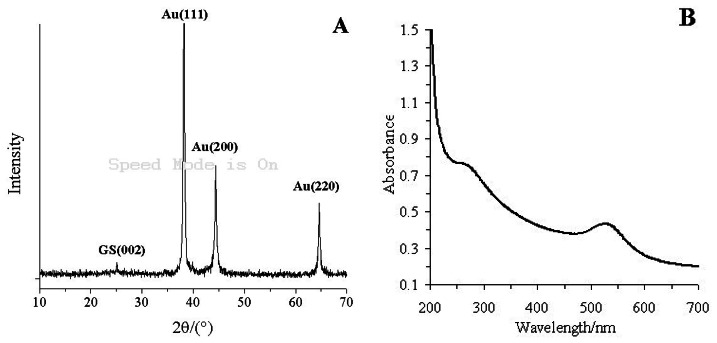
(**A**) The XRD pattern of GS-PEI-Au nanocomposites; (**B**) The UV-vis absorbances pectrum of GS-PEI-Au nanocomposites.

Figure 2A shows the X-ray diffractogram of the GS-PEI-Au nanocomposites. GS has a characteristic peak centered at 2θ = 25.14°, corresponding to the (002) inter-planar spacing of 8.5 Å [36]. The typical diffraction peaks of Au were at 2θ = 38.20°, 44.40° and 64.62°, corresponding to the Au crystal of face-centered cubic (FCC) form, which were composed of the (111), (200) and (220) planes [37]. This indicated that PEI has successfully reduced AuCl_4_^−^ to Au nanoparticles [38].

Figure 2(B) shows the UV-vis absorbance spectra of GS-PEI-Au nanocomposites. GS has an absorption peak at 266 nm, which results from the ð electron electron excitation in the structure of GS [39]. The absorption peaks of GS-PEI-Au nanocomposites observed at 266 and 525 nm correspond to the absorptions of GS and Au nanocomposites [40]. The XRD pattern and UV-vis absorbance spectrum demonstrated that the GS-PEI-Au nanocomposites were prepared successfully.

**Figure 3.**
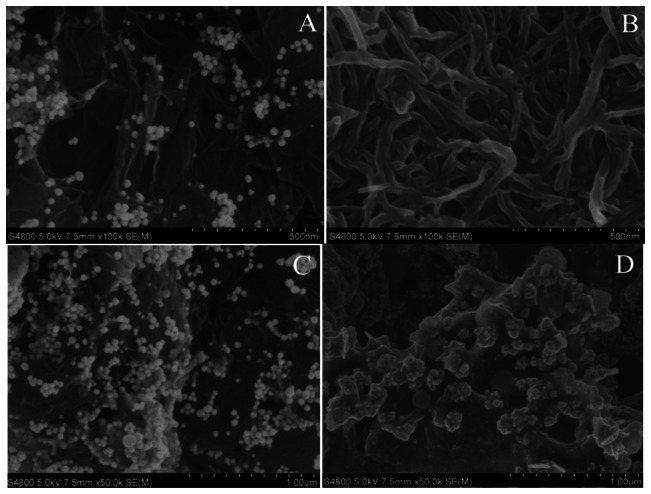
The SEM images of (**A**) GS-PEI-Au nanocomposites; (**B**) MWCNTs/GCE; (**C**) GS-PEI-Au/MWCNTs/GCE; and (**D**) AuNPs-Ab/GS-PEI-Au/MWCNTs/GCE.

The morphologies of Au nanoparticles attached GS was confirmed by the SEM images (Figure 3(A)). It was clearly shown that Au nanoparticles were anchored around the surface of the GS. We assumed that the presence of GS catalyzes the reduction of HAuCl_4_ to Au, which made the reduction of HAuCl_4_ occur on the GS surface.

Figure 3(B) is the image of MWCNTs/GCE. As shown in Figure 2(B), MWCNTs were evenly embedded and well dispersed within the chitosan matrix, which was attributed to the good solubility and dispersing ability of chitosan.

Figure 3(C) shows the morphologies of GS-PEI-Au/MWCNTs/GCE. The GS-PEI-Au nano-composites were firmly and uniformly adsorbed through strong electrostatic interactions, and the Au nanoparticles were scattered on the surface of the GS. Furthermore, this uniform nanostructure provided an efficient surface for loading AuNPs-Ab conjugate and accelerating electron transfer.

Figure 3(D) is the image of AuNPs-Ab/GS-PEI-Au/MWCNTs/GCE. After coating with AuNPs-Ab conjugate, the GS-PEI-Au/MWCNTs/GCE surface became dim and the addition of AuNPs-Ab conjugate resulted in dramatic aggregation. This confirmed that AuNPs-Ab conjugate had been successfully adsorbed on the electrode surface.

### 3.2. Electrochemical Characterization of the Modification Process

**Figure 4.**
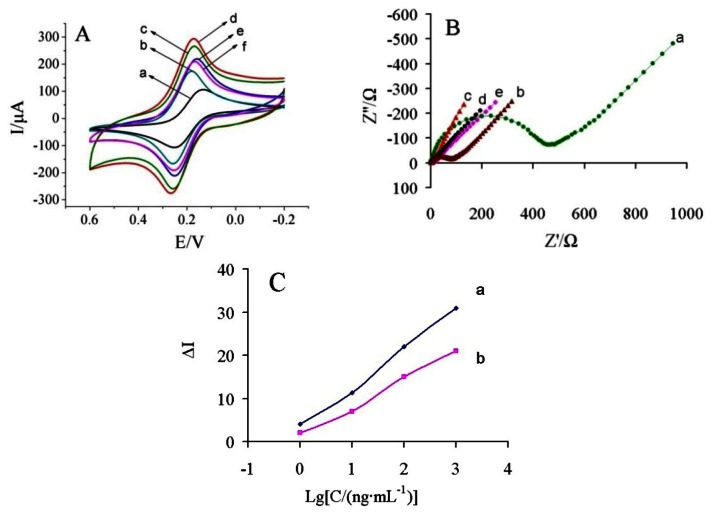
CVs of (**A**): (a) bare GCE, (b) MWCNTs/GCE, (c) GS-PEI-Au/GCE, (d) GS-PEI-Au/MWCNTs/GCE, (e) BSA/AuNPs-Ab/GS-PEI-Au/MWCNTs/GCE, and (f) carbofuran/BSA/AuNPs-Ab/GS-PEI-Au/MWCNTs/GCE; EIS of (**B**): (a) bare GCE, (b) MWCNTs/GCE, (c) GS-PEI-Au/MWCNTs/GCE, (d) BSA/AuNPs-Ab/GS-PEI-Au/ MWCNTs/GCE, and (e) carbofuran/BSA/AuNPs-Ab/GS-PEI-Au/MWCNTs/GCE; (**C**) Amperometric responses of the immunosensor with the various antibodies toward different carbofuran concentrations: (a) AuNPs-Ab conjugate, (b) anti-carbofuran Ab. All of these curves were obtained in 5.0 mM [Fe(CN)6]^3−/4−^ (pH 7.0) containing 0.1 M KCl at 50 mV/s.

Figure 4(A) shows the CVs of the modification process in 5 mM [Fe(CN)_6_]^3−/4−^ solution containing 0.1 M KCl. At the bare GCE, a couple of redox peaks was observed with a peak-to-peak separation (ΔEp) of 0.117 V (curve a). When the electrode was coated with MWCNTs, a decrease of 0.078 V in ΔEp and an increase in peak current (Ip) were obtained (curve b), indicating that MWCNTs could promote the electron transfer between the electrode surface and [Fe(CN)6]^3−/4−^. In contrast, when GS-PEI-Au nanocomposite was immobilized onto the GCE surface, the ΔEp decreased to 0.088 V and the Ip increased sharply (curve c). When MWCNTs and GS-PEI-Au were immobilized gradually onto GCE, the ΔEp of GS-PEI-Au/MWCNTs/GCE was 0.092V and the Ip increased obviously (curve d) compared with MWCNTs/GCE and GS-PEI-Au/GCE, which could be attributed to the synergy of MWCNTs and GS-PEI-Au. This reasonably indicated that MWCNTs and GS-PEI-Au play an important role similar to conducting wire and have the ability to promote the electron transfer. Thus, the response of the immunosensor was improved significantly. Therefore, we fabricated an amperometric immunosensor based on MWCNTs and GS-PEI-Au nanocomposites in this paper. When AuNPs-Ab conjugate and BSA (curve e) were immobilized on the electrode surface, the peak currents decreased obviously. After carbofuran molecules were combined with the Ab, a decrease of the redox peaks was observed (curve f).

EIS of the electrodes were performed in a background solution of 5 mM [Fe(CN)6]^3−/4−^ solution containing 0.1 M KCl, and the frequency range was at 100 mHz to 100 kHz at 200 mV. Figure 4(B) shows the Nyquist diagram of EIS corresponding to the stepwise modification processes. There was a large semicircle (Ret = 234 Ω) at high frequencies and a linear part at low frequencies in the EIS of the bare GCE (curve a). When MWCNTs was modified onto the GCE surface, a significant lower resistance was obtained (curve b, Ret = 39.79 Ω), implying that the MWCNTs was an excellent electron conducting material and accelerated the electron transfer. Furthermore, the GS-PEI-Au/MWCNTs/GCE showed just a straight line (curve c), indicating that the introduction of the GS-PEI-Au was highly beneficial to the electron transfer. This result demonstrated that GS-PEI-Au nanocomposites have been successfully assembled onto the electrode surface. When AuNPs-Ab conjugate and BSA were immobilized onto the GS-PEI-Au/MWCNTs/GCE surface, the Ret increased to 4.71 Ω (curve d). This reason was that the antibody formed an insulating layer on the electrode surface, leading to a higher electron transfer resistance, which was caused by the nonconductive properties of the biomacromolecules. Finally, when the immunosensor was used to detect carbofuran, the Ret increased to 5.59 Ω (curve e).

### 3.3. Comparison of Electrochemical Response

In order to clarify the advantage of AuNPs-Ab conjugation, Ab and AuNPs-Ab conjugate was immobilized onto the GS-PEI-Au/MWCNTs/GCE, respectively. As shown in Figure 4(C), the immunosensor using AuNPs-Ab conjugate exhibited a higher sensitivity than pure Ab. Some possible explanations might be as follows: (i) AuNPs can further amplify the specific surface area, which has good conductivity and biocompatibility to maintain the bioactivity of the Ab. (ii) AuNPs-Ab conjugation provided a stable and covalent immobilization of the Ab with free antigen binding sites and enhanced the sensitivity of the immunoassay. Therefore, the proposed immunosensor could display better analytical properties under the same conditions.

### 3.4. Optimization of Analytical Conditions

**Figure 5.**
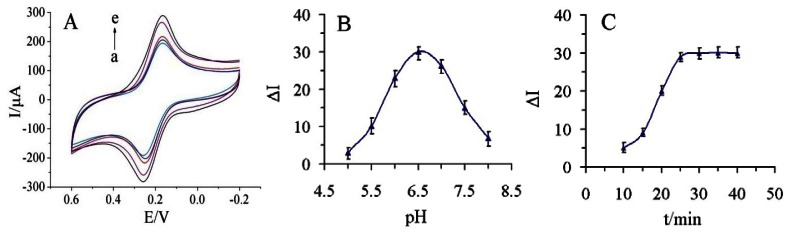
Effect of the thickness of the GS-PEI-Au layer (**A**) (from a to e: 2 μL, 4 μL, 6 μL, 8 μL, 10 μL), the pH of the detection solution (**B**) and the incubation time (**C**) on the immunosensor response in 5.0 mM [Fe(CN)6]^3−/4−^ (pH 7.0) containing 0.1 M KCl.

The parameters on the detection methid, including the thickness of the GS-PEI-Au layer, the pH of the supporting electrolyte, immunoassay temperature and incubation time, were all considered. The thickness of GS-PEI-Au layer would improve the conductivity of the electrode and increase the absorbance of AuNPs-Ab conjugate, so the thickness of the GS-PEI-Au layer greatly affected the analytical performance of the proposed immunosensor. As shown in Figure 5(A), the response current increased with increasing addition of GS-PEI-Au nanocomposite suspension from 2 μL to 10 μL. Considering the area of GCE we used in this study, further addition (>10 μL) could not load on the surface of GCE. Thus, 10 μL of GS-PEI-Au was added on MWCNTs/GCE in our study.

The investigation of different pH values of the supporting electrolyte was performed in series of 0.1 M PBS containing 5.0 mM [Fe(CN)6]^3−/4−^ and 0.1M KCl with the pH from 5.0 to 8.0 (Figure 5(B)). As shown in Figure 5(A), the highest response was found at pH 6.5, and the response then decreased at pH values above 7.0.

Although 37 °C was better for immunoreaction, a high temperature may damage the multilayer structure of GS-PEI-Au nanocomposites, so the final practical operating temperature was chosen to be 25 °C. Finally, the incubation time was chosen based on the effective decrease of current after the immunoreaction. As shown in Figure 5(C), the decrease in current increased within 25 min and then reached a plateau. Thus, the optimum incubation time was set at 25 min for the incubation steps in this study.

### 3.5. Current Response of Immunosensor to Carbofuran Concentration

**Figure 6.**
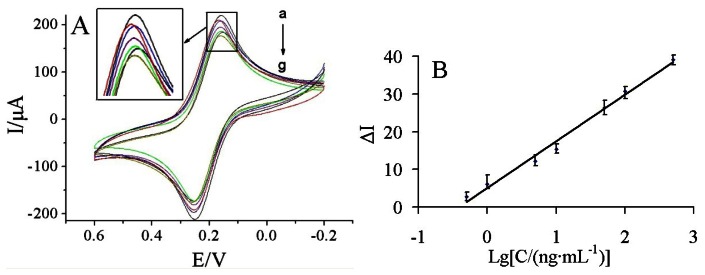
(**A**) The CVs responses of the fabricated immunosensor to the different concentration of carbofuran (from a to g): 0.5, 1, 5, 10, 50, 100, 500 ng/mL under the optimal conditions; (**B**) The calibration curve of the ΔI of the proposed immunosensor *vs.* the logarithm of carbofuran concentration.

Figure 6(A) shows the current response of the immunosensor incubated with various concentrations of carbofuran under the optimal conditions.

It was found that the current response decreased with increasing carbofuran concentrations. It may be due to more carbofuran binding to the immobilized antibodies at higher carbofuran concentrations, which acts as a barrier for the electron transfer. A linear relationship between the ΔI and logarithm of carbofuran solution was obtained in the range of 0.5–500 ng/mL (Figure 6(B)). The linear regression equation was ΔI = 5.155 + 12.325 lgC (ng/mL), with a correlation coefficient of 0.9898. The detection limit was estimated to be 0.03 ng/mL at a signal/noise of 3 (S/N = 3) between the detection signal of low concentration samples and the noise of blank samples.

The performance of the prepared immunosensor was compared with other reported sensors for the detection of carbofuran. As shown in Table 1, compared with other methods, the immunosensor has a relative large linear range and lower detection limit.

### 3.6. Reproducibility, Stability, Selectivity and Regeneration of the Immunosensor

To evaluate the reproducibility of the immunosensor, five electrodes were prepared for the detection of 100 ng/mL carbofuran. The relative standard deviation (RSD) of the measurements for the five electrodes was 3.6%, suggesting the precision and reproducibility of the proposed immunosensor was quite good.

The prepared immunosensors were stored at 4 °C for four weeks when not in use and the current responses of the stored immunosensors retained over 89% of their original value, indicating that the current immunosensor maintained its immunoactivity over long-term storage and an acceptable stability was obtained. The probable reasons can be attributed to the following factors: firstly, the synergy of MWCNTs and GS-PEI-Au nanocomposites effectively maintained the stability of the immunosensor. Secondly, AuNPs have good conductivity and biocompatibility to maintain the bioactivity of the Ab.

**Figure 7.**
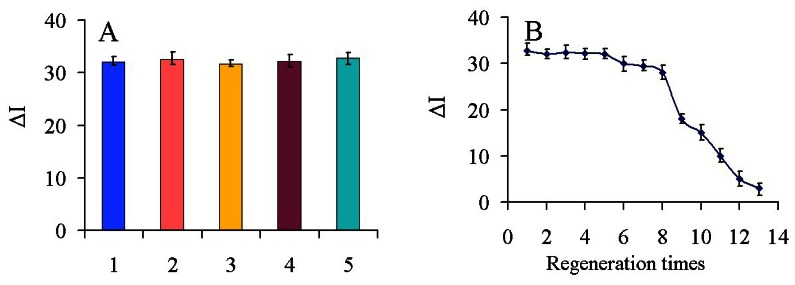
(**A**) The ΔI of proposed immunosensor to: (1) 100 ng/mL carbofuran, (2) 100 ng/mL carbofuran+100 ng/mL chlorpyrifos, (3) 100 ng/mL carbofuran+100 ng/mL dichlorphos, (4) 100 ng/mL carbofuran+100 ng/mL 3-hydroxycarbofuran, (5) 100 ng/mL carbofuran+100 ng/mL carbaryl; (**B**) Regeneration performance of the immunosensor.

To investigate the selectivity of the fabricated immunosensor, interference studies were performed using chlorpyrifos, dichlorphos, 3-hydroxycarbofuran and carbaryl which are commonly present in real samples. A mixed solution of 100 ng/mL carbofuran containing 100 ng/mL of the other above-mentioned pesticides (1:1) was measured with the immunosensor and the results are shown in Figure 7(A). The current variation due to the interference was less than 4% of the current response in the absence of interference, indicating that the selectivity of the immunosensor was acceptable.

To test the possibility of regenerating the immunosensor, 0.1 M glycine-HCl buffer (pH 2.8) was used to wash the electrode. After the detection of 100 ng/mL carbofuran, the immunosensor was immersed into the glycine-HCl buffer for 5 min to break the hapten-antibody linkage. As shown in Figure 7(B), after eight regeneration cycles, the immunosensor retained about 90% of its original value, and a RSD of 5.1% was obtained. This is likely because Ab can gradually shell off or denature and the structure of nanocomposites can be destroyed during continuous processing by a glycine-HCl buffer and cleaning with the increase of regeneration times. Therefore, it affected the binding activities and capacities between Ab and carbofuran. The results showed that the immunosensor had a good regeneration performance and could be regenerated up to eight times.

### 3.7. Analysis of Real Samples

In order to evaluate the feasibility of the using immunosensor for possible applications, the proposed immunosensor was used to determine the recoveries of different concentrations of carbofuran in several real samples, including cabbages, green peppers, tomatoes, Chinese chives and peaches. Each sample was prepared as follows and analyzed five times: (1) the samples were washed and dried; (2) the samples were cut up into 3 × 3 mm pieces; (3) 1.0 g samples and the same volume of carbofuran were added to three tubes, tightly stoppered and mixed for 12h; (4) 5 mL PBS was added into these three tubes and then sonicated for 30 min; (5) after being sonicated, the obtained solution was cooled down to room temperature and filtered through a 0.22 μm filter membrane. The resulting solution was used as the sample solution. Another three tubes were prepared using the same operation methodology mentioned above not real samples were not added. The resulting solution was used as the reference solution. The results showed that the RSD was between 2.33% and 4.53% and the average recovery was in the range of 86.0%–103.0% (Table 2). Hence, the developed immunosensor could be satisfactorily applied to the direct analysis of carbofuran in real samples.

## 4. Conclusions

In this work, a label-free amperometric immunosensor for the rapid detection of carbofuran has been successfully developed and applied. The synergy effect of MWCNTs and GS-PEI-Au nanocomposites and the effect of covalent immobilization of AuNPs-Ab conjugate have been investigated, respectively. Because MWCNTs and GS-PEI-Au nanocomposites can greatly enhance the electron transfer between the electrolyte and electrode, as well as increase the surface area to capture a large amount of Ab. More significantly, AuNPs-Ab conjugate was prepared for immobilizing Ab more efficiently, which would be useful to provide a stable and covalent immobilization of the Ab with the free hapten binding sites. This strategy can be observed to improve the immunoassay sensitivity, and thus provides a novel promising platform of immunoassay for carbofuran residues detection.

## Figures and Tables

**Table 1.** Comparison of analytical methods for the detection of carbofuran.

**Electrode Modification**	**Linear Range (ng/mL)**	**Detection Limit (ng/mL)**	**Reference**
ELISA	-	25	[41]
AuNP/AChE/Au electrode	-	7.293	[42]
AChE/PAMAN-Au/CNTs/GCE	1–20	0.89	[43]
AChE/TCNO/SPE	0.2–166	0.2	[44]
BSA/AuNPs-Ab/GS-PEI-Au/ MWCNTs/GCE	0.5–500	0.03	This work

**Table 2.** The recovery of the proposed immunosensor in real samples.

**Sample**	**Added (ng/mL)**	**Found (ng/mL)**	**RSD (%) (n=5)**	**Recovery (%)**
Lettuce	1.0	0.91	3.48	91.0
	1.0 × 10^2^	0.89 × 10^2^	3.26	89
	5.0 × 10^2^	5.06 × 10^2^	2.64	101.2
Cabbage	1.0	0.86	3.15	86.0
	1.0 × 10^2^	0.93 × 10^2^	3.68	93.0
	5.0 × 10^2^	5.03 × 10^2^	4.11	100.6
Green peppers	1.0	0.95	2.87	95.0
	1.0 × 10^2^	0.96 × 10^2^	2.48	96.0
	5.0 × 10^2^	5.05 × 10^2^	2.6	101.0
Tomatoes	1.0	1.03	2.33	103.0
	1.0 × 10^2^	0.98 × 10^2^	3.61	98.0
	5.0 × 10^2^	4.89 × 10^2^	4.53	97.8
Chinese chives	1.0	0.86	4.22	86.0
	1.0 × 10^2^	1.02 × 10^2^	4.03	102.0
	5.0 × 10^2^	4.88 × 10^2^	2.59	97.6
Peaches	1.0	0.92	3.94	92
	1.0 × 10^2^	0.90 × 10^2^	2.66	90
	5.0 × 10^2^	4.87 × 10^2^	4.05	97.4
